# Integrated Ultrastructural and Transcriptomic Analysis of the Proboscis in *Apis cerana cerana* Under Different Foraging Conditions

**DOI:** 10.3390/ani16182936

**Published:** 2026-09-18

**Authors:** Chang Song, Yu Zhang, Lina Guo, Yuan Guo

**Affiliations:** 1 College of Animal Science, Shanxi Agricultural University, Jinzhong 030801, China; 2 Shanxi Key Laboratory of Animal Nutrition and Feed Development, College of Animal Science, Shanxi Agricultural University, Jinzhong 030801, China; 3 College of Horticulture, Shanxi Agricultural University, Jinzhong 030801, China

**Keywords:** *Apis cerana cerana*, proboscis, scanning electron microscopy (SEM), transcriptome, differentially expressed genes, odorant receptor (OR)

## Abstract

The proboscis of *Apis cerana cerana* is primarily regarded as a feeding organ, whereas its potential structural and molecular characteristics associated with chemosensory processes remain poorly understood. This study integrated ultrastructural and transcriptomic analyses to investigate the morphological characteristics and chemosensory-associated molecular features of the proboscis under different pollen-load conditions. Scanning electron microscopy revealed a highly organized glossal architecture characterized by longitudinal ridge–groove structures and densely distributed hair-like structures on the distal glossa and flabellum, indicating regional structural specialization of the proboscis surface. Transcriptomic analyses identified 1549 and 1652 differentially expressed genes (DEGs) in workers carrying pear pollen loads and rapeseed pollen loads, respectively, compared with workers without visible pollen loads, whereas only 89 DEGs were detected between the two pollen-load groups. These results indicate that pollen-load status was associated with greater transcriptional differences than the comparison between the two floral sources under the present experimental conditions. Functional enrichment analyses showed that DEGs were mainly associated with cellular processes, signal transduction, and metabolic regulation pathways. Additionally, six candidate OR-like transcripts, including several odorant receptor-like genes, were identified from the proboscis transcriptome, and their expression patterns were validated by quantitative real-time PCR. Phylogenetic analyses revealed both conserved orthologs and lineage-specific divergence. Collectively, these structural and molecular findings suggest that the honey bee proboscis may possess chemosensory-related characteristics, providing a basis for future studies investigating their functional significance.

## Simple Summary

The proboscis of the eastern honey bee, *Apis cerana cerana*, is traditionally considered a feeding organ used for nectar collection. However, its potential structural and molecular characteristics associated with chemical sensing during foraging remain insufficiently understood. In this study, we combined scanning electron microscopy and transcriptome sequencing to investigate the structural and molecular characteristics of the proboscis under different foraging conditions. Ultrastructural observations revealed abundant hair-like sensilla and specialized ridge–groove structures on the glossa and flabellum, suggesting possible specialization of the proboscis surface for interactions with external stimuli. Transcriptome analyses showed substantial differences in gene expression between pollen-load groups and bees without visible pollen loads, while differences between the two pollen sources were relatively limited under the present experimental conditions. Several candidate OR-like transcripts, including odorant receptor-like genes, were identified in the proboscis transcriptome, and phylogenetic analyses revealed conserved and lineage-specific evolutionary patterns among candidate sequences. These findings provide preliminary evidence that the honey bee proboscis contains structural and molecular features potentially associated with chemosensory processes, although further functional studies are required. This study provides new insights into the potential involvement of the honey bee proboscis in sensory-related processes during foraging behavior.

## 1. Introduction

As typical social pollinating insects, honey bees serve as key agents of pollination in terrestrial ecosystems. The precise regulation of their foraging and pollination behaviors plays an irreplaceable role in maintaining ecological balance and ensuring agricultural productivity [1]. *Apis cerana cerana*, an indigenous and ecologically dominant honey bee subspecies in Asia, can exploit dispersed floral resources across different latitudes, altitudes, and climatic conditions. It maintains stable foraging activity in diverse and complex natural environments [2,3]. Through long-term ecological adaptation, *Apis cerana cerana* has evolved a highly refined chemosensory system, in which olfaction plays an important role in recognizing floral odor cues, navigating foraging routes, and regulating foraging-related behavior. In insects, antennal odorant receptors (ORs) and their obligate co-receptor Orco form the core molecular components underlying volatile odor recognition, contributing to sensitivity toward odor cues and associated behavioral responses [4,5,6,7]. Although existing studies on honey bee olfaction have mainly focused on the antennal system, chemical cues may also influence bee behavior during close-range interactions with flowers and feeding activities.

The proboscis, as the anterior feeding organ, extends deep into the corolla and directly contacts nectar during feeding [8,9]. This position enables the proboscis to be exposed to floral volatiles at close range while being continuously exposed to the localized odor environment formed by nectar-derived volatile components [10,11,12]. The proboscis surface may represent more than a simple feeding structure. Its finely partitioned ultrastructural regions and the distribution of multiple types of sensilla [2], particularly the presence of apical pores or openings on certain sensilla [13], provide structural features that may facilitate interactions between external chemical molecules and sensory structures. These morphological characteristics suggest that the proboscis contains structural features that may contribute to chemical cue detection during feeding [14,15]. Together, these sensilla may contribute to the sensory functions of the honey bee proboscis by facilitating the detection of sugars, amino acids, and other chemical components in nectar, thereby potentially influencing feeding preference and choice behavior [16]. However, current studies on honey bee chemosensory mechanisms have focused on antennal tissues, whereas molecular characteristics of mouthpart-associated chemosensation remain less explored. Systematic and in-depth analyses of OR expression patterns in mouthparts, particularly in the proboscis, under different foraging-related conditions remain limited [17]. Given that floral volatile compositions differ substantially among plant species, bees exposed to different floral resources may exhibit variation in molecular responses related to chemical perception, potentially resulting in tissue-specific transcriptional changes [18]. Previous studies have suggested that honey bee mouthparts may possess sensory characteristics involved not only in gustatory perception but may also participate in detecting localized chemical cues during feeding. However, direct molecular evidence supporting potential volatile-sensing capability in the proboscis remains limited, and integrated analyses combining ultrastructure and transcriptomics are still lacking [19]. Therefore, this study aimed to investigate whether chemosensory-related OR-like transcripts are expressed in the proboscis of *Apis cerana cerana* and whether their expression patterns differ among workers with different pollen-load conditions. In this study, scanning electron microscopy (SEM) and transcriptome sequencing were integrated to systematically characterize the ultrastructural features of the *Apis cerana cerana* proboscis, with a specific focus on the morphology and distribution of surface structures and sensilla. Based on these morphological findings, there are three pollen-load groups—workers carrying pear pollen, workers carrying rapeseed pollen, and workers without visible pollen loads. An integrated analysis of both morphological and molecular expression data was subsequently performed. By integrating ultrastructural observations with transcriptomic analyses, this study aims to provide preliminary structural and molecular evidence supporting the potential involvement of the proboscis in chemical cue-associated processes during feeding. Furthermore, it aims to provide novel insights into potential coordination of sensory mechanisms across multiple honey bee tissues.

## 2. Materials and Methods

### 2.1. Experimental Insects

Experimental colonies of *Apis cerana cerana* were collected from 23 to 29 March 2022, within an ecological pear orchard located in Hongzhiyi Town, Yanhu District, Yuncheng City, Shanxi Province, China (Longitude: 110.99827°, Latitude: 35.0151°). The orchard encompassed approximately 54 hectares and was predominantly cultivated with pear trees. A 0.2-hectare rapeseed field, exhibiting a competing plant proportion of 0.4%, was situated 1000 m away from the pear orchard. Owing to this uneven landscape configuration, foragers collecting rapeseed pollen (YH group) represented a small subset of the local foraging population, and bees from the YH and LH groups were exposed to substantially different surrounding floral backgrounds during foraging. This landscape-derived confounding factor was unavoidable under our field sampling design. Each biological replicate was generated by pooling proboscises from workers collected from multiple colonies. Thus, each biological replicate represented a pooled sample derived from multiple colonies. The bee colonies were maintained in six Langstroth hives, each housing an estimated 3000–3500 worker bees, with efforts made to maintain consistent population sizes. Each hive contained a single queen, a small number of larvae, and limited reserves of pollen and nectar. When approximately 25% of the pear blossoms had opened, the colonies were relocated to the orchard, and the hives were strategically positioned in a single row between the pear trees and the rapeseed field.

From 11:00 a.m. to 3:00 p.m. on the subsequent day, a total of 600 worker bees were collected, with 200 workers assigned to each experimental group (LH, YH, and CH). These bees were subsequently assigned to three experimental groups based on the presence and apparent botanical origin of their pollen loads: workers carrying pear-associated pollen loads (LH), workers carrying rapeseed-associated pollen loads (YH), and workers without visible pollen loads (CH). The apparent botanical origin of pollen loads was classified according to visible pollen pellet color characteristics and the dominant flowering plants surrounding the apiary. Rapeseed-associated pollen loads were generally characterized by a bright yellow appearance, whereas pear-associated pollen loads typically exhibited a pale yellow to light brown coloration under field observation. Therefore, specific pollen loads were used as a practical indicator of recent visits to different floral resources, rather than as definitive evidence that the proboscis had been exclusively exposed to the nectar, volatile compounds, or other localized chemical cues from those plant species.

The proboscises were dissected under icy conditions, immediately flash-frozen in liquid nitrogen, and stored at −80 °C until further use. Approximately 600 worker bees were initially collected to ensure sufficient material for subsequent tissue dissection and experimental screening. From these individuals, proboscis tissues meeting the experimental criteria were selected for SEM observation, RNA-seq, and qRT-PCR analyses.

To minimize environmental variation, all sampled colonies were maintained within the same apiary under similar management conditions. Colonies were selected to have comparable population sizes and nutritional states. Worker bees carrying visible pollen loads were classified as pollen-collecting workers, while those collected at the hive entrance without visible pollen loads served as the comparison group. It should be noted that the absence of visible pollen loads does not necessarily indicate a lack of foraging activities; this group likely included nectar foragers, water collectors, or scout bees. Therefore, this comparison primarily reflects differences associated with pollen-foraging status, rather than a strict distinction between foraging and non-foraging individuals. 

Workers were collected from multiple colonies maintained in the same apiary. For RNA-seq analysis, three biological replicates were prepared for each experimental group (LH, YH, and CH). A total of 600 worker bees were collected, with 200 workers assigned to each experimental group (LH, YH, and CH). Individuals meeting the pollen-load criteria were randomly selected for subsequent SEM, RNA-seq, and qRT-PCR analyses. Therefore, biological replicates represented independent colony-derived samples rather than multiple individuals from the same colony.

### 2.2. Scanning Electron Microscopy (SEM) Sample Preparation and Observation

Healthy worker bees were selected for SEM analysis, and proboscises from seven worker bees were examined to characterize the external ultrastructural features. Their proboscises were carefully dissected under a stereomicroscope using fine forceps or a scalpel to preserve structural integrity and surface morphology. The isolated proboscises were fixed in a 2.5–3.0% glutaraldehyde solution and subsequently rinsed 3–5 times with 0.1 M phosphate buffer (PB) to remove residual fixative. Specimens were subsequently rinsed repeatedly with phosphate-buffered saline (PBS) to remove residual fixative. Dehydration was performed sequentially through graded ethanol solutions of 30%, 50%, 70%, 80%, 90%, 95%, and 100%, with each step lasting 5–15 min to ensure gradual replacement of intracellular water. Dehydrated specimens were then subjected to critical-point drying using a JFD-320 critical-point dryer (JEOL, Tokyo, Japan). Following drying, samples were coated with a thin conductive layer (approximately 10 nm) of gold or gold–palladium alloy using an SBC-12 sputter coater (KYKY Technology Co., Ltd., Beijing, China). This procedure enhanced sample conductivity and minimized charge accumulation, thereby facilitating stable imaging during SEM observation.

The prepared samples were mounted on the stage of a JSM-7800F (JEOL Ltd., Akishima, Tokyo, Japan) scanning electron microscope. The accelerating voltage was optimized (5–20 kV) according to observed structural characteristics and resolution requirements. Images were captured at various magnifications to document the surface morphology, surface structures and sensilla distribution of the proboscis. Acquired images were not subjected to brightness, contrast, or other adjustments that could alter structural information.

### 2.3. RNA Extraction, Library Construction, and Transcriptome Sequencing

Total RNA was extracted using TRIzol reagent (Thermo Fisher Scientific, Cat. No. 15596018, Waltham, MA, USA) in accordance with the manufacturer’s protocol. RNA concentration and purity were assessed via an Agilent 2100 Bioanalyzer equipped with the RNA 6000 Nano Kit (Agilent Technologies, Santa Clara, CA, USA; Cat. No. 5067-1511). RNA samples exhibiting an RNA integrity number (RIN) > 7.0 were selected for subsequent library construction. The RNA samples used for transcriptome sequencing were derived from the same biological groups used for subsequent qRT-PCR validation.

After RNA extraction, 5 μg of total RNA was used for mRNA isolation through a two-step purification process utilizing oligo (dT) magnetic beads (Thermo Fisher Scientific, Waltham, MA, USA). Purified mRNA was fragmented into short segments by incubation at 94 °C for 5–7 min using a magnesium RNA fragmentation kit (New England Biolabs, Ipswich, MA, USA; Cat. No. E6150), wherein divalent cations induced fragmentation.

The fragmented RNA was reverse-transcribed into cDNA using Reverse Transcriptase II (Invitrogen, Waltham, MA, USA; Cat. No. 1896649). Subsequently, uracil-labeled double-stranded cDNA was synthesized using *Escherichia coli* DNA Polymerase I (New England Biolabs, Ipswich, MA, USA; Cat. No. M0209), RNase H (New England Biolabs, USA; Cat. No. M0297), and dUTP solution (Thermo Fisher Scientific, Waltham, MA, USA; Cat. No. R0133).

The double-stranded cDNA underwent end repair and A-tailing to facilitate the ligation of indexed sequencing adapters. Each adapter incorporated a T-base overhang to ensure efficient ligation with the A-tailed DNA fragments. Dual-index adapters were ligated to the DNA fragments, followed by fragment size selection using AMPure XP magnetic beads (Beckman Coulter, Inc., Indianapolis, IN, USA). After treatment of the uracil-labeled double-stranded cDNA with heat-labile uracil-DNA glycosylase (New England Biolabs, USA; Cat. No. M0280), the ligation products were amplified via PCR under the following conditions: initial denaturation at 95 °C for 3 min; 8 cycles of 98 °C for 15 s, 60 °C for 15 s, and 72 °C for 30 s; followed by a final extension at 72 °C for 5 min. The final cDNA libraries had an average insert size of 300 ± 50 bp.

Sequencing was performed by Hangzhou Lianchuan Biotechnology Co., Ltd. (Hangzhou, China) on the Illumina NovaSeq™ 6000 platform (Illumina, Inc., San Diego, CA, USA), generating paired-end 150 bp reads (PE150) as per the manufacturer’s standard protocol. The raw sequencing data have been deposited in the NCBI Gene Expression Omnibus (GEO) database under accession number GSE324143.

### 2.4. RNA-Seq Data Processing and Bioinformatics Analysis

Raw sequencing data were initially subjected to quality control using Cutadapt v1.9 with default parameters. This step was performed to remove sequencing adapter contamination, polyA/T and polyG tails, low-quality reads (containing >20% bases with Q ≤ 20), and reads containing >5% ambiguous bases (N). Following filtering, sequence quality was further assessed using FastQC software (http://www.bioinformatics.babraham.ac.uk/projects/fastqc, accessed on 15 April 2026, version 0.11.9). The quality metrics evaluated included Q20 values, Q30 values, and GC content of the clean reads.

High-quality clean reads from the nine libraries were aligned to the *Apis cerana cerana* reference genome (GCF_001442555.1) using HISAT2 v2.2.1 with default parameters. These parameters allowed up to two mismatches per read and reported up to 20 alignments per read. The aligned reads were then assembled into transcripts using StringTie v2.1.6 (stringtie-2.1.6). Transcript assemblies from all samples were merged using gffcompare v0.9.8 (gffcompare-0.9.8) to generate a comprehensive reference transcriptome. Gene expression levels were quantified using StringTie v2.1.6 and Ballgown R package v2.18.0 and expressed as FPKM (Fragments Per Kilobase of transcript per Million mapped reads).

Functional annotation was performed using BLASTx (BLAST+ v2.12.0) by aligning assembled transcripts against the NCBI non-redundant (NR) database with an E-value threshold of 1 × 10^−5^. Gene Ontology (GO) annotation was conducted using Blast2GO v5.2, classifying gene functions into three categories: biological process, molecular function, and cellular component. KEGG pathway annotation was performed using the KEGG Automatic Annotation Server (KAAS, 2021 version), mapping genes to biochemical and signaling pathways. GO and KEGG enrichment analyses of differentially expressed genes were performed using the hypergeometric test. For GO enrichment analysis, all genes with GO annotations were used as the background gene set, whereas all genes with KEGG annotations were used as the background gene set for KEGG pathway enrichment analysis. Multiple testing correction was performed using the Benjamini–Hochberg false discovery rate (FDR) method, and terms with adjusted *p*-values (FDR) < 0.05 were considered significantly enriched.

### 2.5. Identification of Differentially Expressed Genes

To accurately identify significant changes in gene expression among different pollen-load groups, statistical comparisons were conducted between each pair of experimental groups. Differentially expressed genes (DEGs) were identified using DESeq2 v1.30.1 based on raw read counts from biological replicates. Genes with a |log2 fold change| ≥ 1 and an adjusted *p*-value (false discovery rate, FDR) < 0.05 were considered significantly differentially expressed.

For DEG identification, raw read counts mapped to each gene were obtained using HTSeq and used as input for DESeq2 analysis. FPKM values were calculated separately for expression-level visualization and comparison among samples and were not used for differential expression analysis. Several variables, including worker age, behavioral specialization, and individual foraging history, were not experimentally controlled in this study. Additionally, the group without visible pollen loads may have included a heterogeneous mix of nectar foragers, water collectors, and scout bees. Consequently, the identified transcriptional differences should be interpreted cautiously; they represent associations with pollen-load conditions rather than direct evidence of floral odor-specific regulation.

### 2.6. Identification of Candidate Odorant Receptor (OR)-like Transcripts 

To identify candidate odorant receptor (OR)-related sequences associated with differential expression patterns in the proboscis transcriptome of *Apis cerana cerana*, differentially expressed genes (DEGs) were screened based on transcriptome annotation results. Candidate OR-related transcript sequences were subsequently analyzed using BLAST against the NCBI database to identify homologous sequences and confirm gene annotations. BLAST analysis identified homologous OR sequences with the highest similarity, together with corresponding gene annotations and accession numbers. Open reading frames (ORFs) of the candidate OR-like transcript sequences were predicted using the NCBI ORF Finder (https://www.ncbi.nlm.nih.gov/orffinder, accessed on 17 April 2026) to identify putative coding regions.

### 2.7. Quantitative Real-Time PCR (qRT-PCR) Validation

To validate the expression patterns of selected candidate odorant receptor (OR) genes identified from the transcriptome analysis, six candidate OR-like transcripts were selected for qRT-PCR analysis. *β-ACTIN*(XM_017059068.3) was used as the internal reference gene. Primer sequences for all target and reference genes are listed in Table 1. Total RNA extracted from proboscis tissues was reverse-transcribed into cDNA, which was subsequently used as the template for amplification. The same RNA samples used for RNA-seq were subsequently used for qRT-PCR analysis to evaluate the consistency of expression trends. qRT-PCR reactions were performed using TB Green^®^ Premix Ex Taq™ II (Cat. No. RR820A, Takara Bio Inc., Kusatsu, Shiga, Japan) under the following conditions: an initial denaturation step at 95 °C for 3 min; followed by 40 cycles of denaturation at 95 °C for 5 s, 57 °C for 30 s, and 72 °C for 30 s; a melting curve analysis was performed after amplification, consisting of 95 °C for 10 s and 52 °C for 5 s. Relative gene expression levels were calculated using the 2^−ΔΔCt^ method. *β-ACTIN* was used as the internal reference gene for normalization. Each treatment group comprised three biological replicates, and each biological replicate consisted of three technical replicates. Melting curve analysis was performed after qRT-PCR amplification. All primer pairs generated a single melting peak, indicating specific amplification without detectable nonspecific products or primer-dimer formation. The average Ct value from the technical replicates was used as the representative Ct value for each sample. ΔCt values were calculated, and statistical analyses were performed on the ΔCt values using one-way ANOVA followed by Tukey’s multiple-comparison test. Relative expression levels were calculated using the 2^−ΔΔCt^ method for visualization. The expression patterns obtained by qRT-PCR were compared with the corresponding RNA-seq results based on the direction and magnitude of log_2_ fold changes. For graphical comparison with RNA-seq results, 2^−ΔΔCt^ values were log_2_-transformed to generate log_2_ (fold-change) values.

### 2.8. Construction of the Odorant Receptor (OR) Phylogenetic Tree

To investigate the phylogenetic relationships between the six candidate AcerORs and odorant receptors from other insects, nucleotide sequences of representative OR-like transcripts from *Apis mellifera* and *Drosophila melanogaster* were selected. These, along with the six candidate AcerORs nucleotide sequences, were used to construct a phylogenetic tree. Representative OR sequences were selected based on their annotation availability and previous characterization in insect olfactory receptor studies. Two separate phylogenetic analyses were conducted to provide complementary evolutionary contexts using a closely related hymenopteran reference, *Apis mellifera*, and a more distantly related insect reference, *Drosophila melanogaster*.

Annotated OR nucleotide sequences of *Apis mellifera* and *Drosophila melanogaster* were retrieved from the NCBI database. A multiple sequence alignment of these downloaded nucleotide sequences and those from *Apis cerana cerana* was performed using MEGA version 12.0. A phylogenetic tree was subsequently constructed using the neighbor-joining (NJ) method implemented in MEGA 12.0 with 1000 bootstrap replicates. Candidate AcerORs were classified according to their phylogenetic relationships with annotated OR sequences. The resulting phylogenetic tree was visualized and refined using iTOL (https://itol.embl.de, accessed on 10 February 2026) for visualization and graphical refinement [20].

## 3. Results

### 3.1. Ultrastructural Characteristics of the Proboscis in Apis cerana cerana 

**Figure 1:**
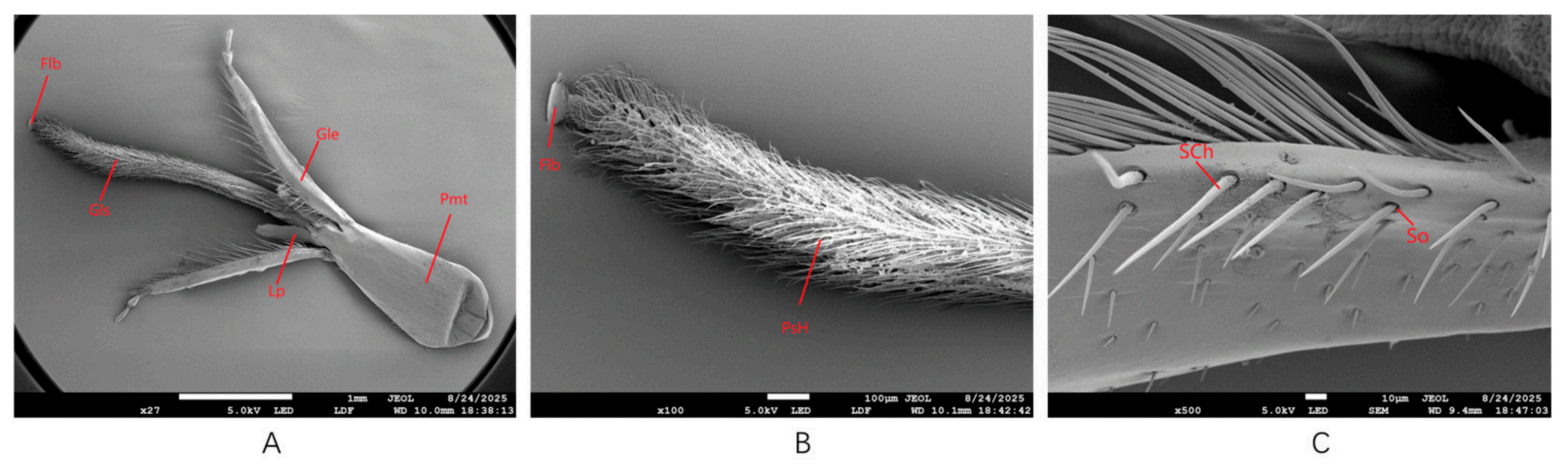
Scanning electron microscopy (SEM) observations of the proboscis morphology of *Apis cerana cerana*. NOTE: (**A**) Overview of the external morphology of the proboscis. (**B**) Enlarged view of the distal glossa showing the flabellum (Flb) and surrounding pseudohairs (PsH). (**C**) Detailed view of the glossal surface showing sensilla chaetica (Sch) located within distinct sockets (So) and distributed among rows of pseudohairs.

SEM observations of seven worker bee proboscises revealed that the proboscis consisted of the prementum (Pmt), glossa (Gls), galeae (Gle), and labial palps (Lp), forming an elongated feeding structure (Figure 1A). The glossa was slender and covered with elongated hair-like structures along its surface, gradually tapering toward the distal end. At the apex of the glossa, a distinct flabellum (Flb) was observed, forming a brush-like structure composed of closely arranged setae. The galeae were positioned lateral to the glossa and extended parallel to it, partially enclosing the proboscis. Setae were distributed along the margins of the galeae. The labial palps originated near the basal region of the proboscis and projected laterally. Compared with the distal regions, the prementum exhibited a relatively smooth surface and formed the enlarged basal supporting region of the proboscis. Overall, the SEM image revealed the spatial organization and external morphology of the major components of the bee proboscis [13].

The distal region of the glossa was covered with elongated pseudohairs (PsH), which were arranged in overlapping rows along the longitudinal axis of the proboscis (Figure 1B). These pseudohairs were slender and curved distally, forming a brush-like surface. Toward the apex of the glossa, the pseudohairs became progressively more compact, ultimately forming the flabellum (Flb), a specialized terminal lobe-like structure at the distal end of the proboscis. The flabellum appeared expanded relative to the adjacent glossal shaft and was composed of tightly packed hair-like projections radiating outward from the apex. The arrangement of pseudohairs greatly contributed to the morphology of the distal glossa. In lateral view, the pseudohairs were oriented obliquely toward the apical direction and partially overlapped one another, producing a feather-like appearance across the glossal surface. The transition between the glossal pseudohairs and the distal flabellum was gradual, without a sharply defined boundary.

SEM observations revealed different types of cuticular projections on the lateral surface of the glossa. Elongated pseudohairs were arranged in parallel rows along the dorsal region of the glossal surface (Figure 1C). These pseudohairs were smooth, slender, and distally oriented, overlapping one another to form a dense brush-like covering. Beneath the pseudohair layer, several larger sensilla chaetica (Sch) were distributed individually along the glossal cuticle. Compared with the surrounding pseudohairs, the sensilla chaetica are thicker and arise from distinct cuticular sockets (So). Each sensillum exhibited a smooth surface and gradually tapers toward a pointed distal tip. The sockets were oval to rounded and slightly recessed into the cuticular surface, providing distinct insertion sites for these sensilla. The sensilla chaetica were irregularly spaced and projected obliquely outward from the glossal surface, extending beyond the surrounding smaller projections. In contrast, the smaller cuticular setae distributed on the ventral region are shorter and more sparsely arranged. The SEM image clearly distinguishes the sensilla chaetica from the densely packed pseudohairs based on their size, orientation, and socket morphology. The surface of the glossa itself appeared relatively smooth between the insertion points of the structures.

### 3.2. Transcriptome Sequencing of the Proboscis in Apis cerana cerana

To characterize gene expression profiles in the proboscis of *Apis cerana cerana* associated with workers classified according to visible pollen-load conditions, transcriptome sequencing was performed using the Illumina sequencing platform. A total of nine cDNA libraries were constructed, including proboscis samples from workers carrying pear-associated pollen loads (LH1, LH2, and LH3), workers carrying rapeseed-associated pollen loads (YH1, YH2, and YH3), and workers without visible pollen loads (CH1, CH2, and CH3). Sequencing quality was evaluated based on clean read proportion, Q20/Q30 values, GC content, and genome mapping rates across all libraries. For the nine libraries (three biological replicates per group: CH, LH, and YH), each sample generated approximately 3.67 × 10^7^ to 5.53 × 10^7^ raw reads. After filtering, approximately 3.54 × 10^7^ to 5.41 × 10^7^ clean reads were obtained, with clean data ratios ranging from 96.42% to 97.82%. The clean reads were subsequently aligned to the *Apis cerana cerana* reference genome. The total mapping rates ranged from 69.80% to 81.45%, while uniquely mapped reads accounted for 68.05% to 79.42% of the clean reads. All samples exhibited Q20 values of 99.98% and Q30 values ranging from 96.48% to 97.20%, with GC contents of approximately 38.5–40.5% (Table 2). These sequencing metrics indicated that the obtained transcriptome data were suitable for subsequent differential expression and functional enrichment analyses. 

### 3.3. Differential Expression Analysis

**Figure 2:**
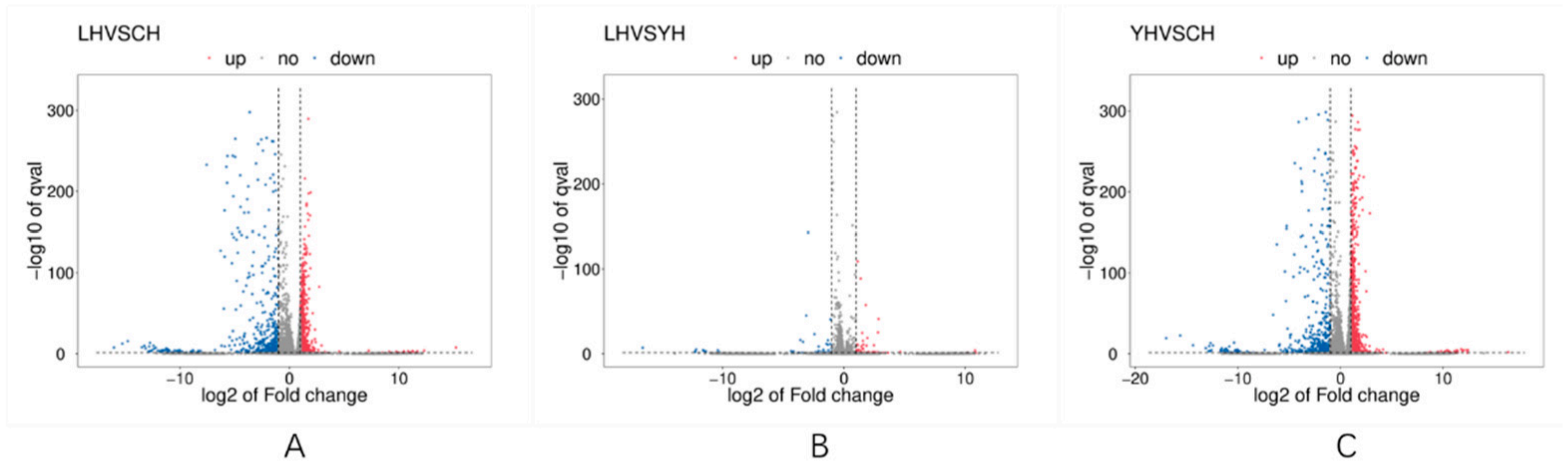
Volcano plots of differentially expressed genes in the proboscis of *Apis cerana cerana* under different pollen-load conditions. Note: (**A**) LH vs. CH groups; (**B**) LH vs. YH groups; (**C**) YH vs. CH groups.

The statistical analysis of differentially expressed genes (DEGs) is shown in Figure 2. In the LH vs. CH comparison, a total of 1549 DEGs were identified, including 1054 upregulated and 495 downregulated genes. In the YH vs. CH comparison, 1652 DEGs were detected, comprising 1143 upregulated and 509 downregulated genes. In contrast, only 89 DEGs were identified in the LH vs. YH comparison, including 43 upregulated and 46 downregulated genes. The substantially greater numbers of DEGs identified in comparisons between pollen-load groups (LH and YH) and workers without visible pollen loads (CH), compared with the relatively small number of DEGs detected between the two pollen-load groups (LH vs. YH), indicate that differences associated with pollen-load conditions were associated with greater transcriptomic variation than differences observed between the two pollen-associated groups under the present experimental conditions. In contrast, fewer transcriptional differences were detected between workers carrying the two different pollen-associated loads under the conditions examined in this study.

**Figure 3:**
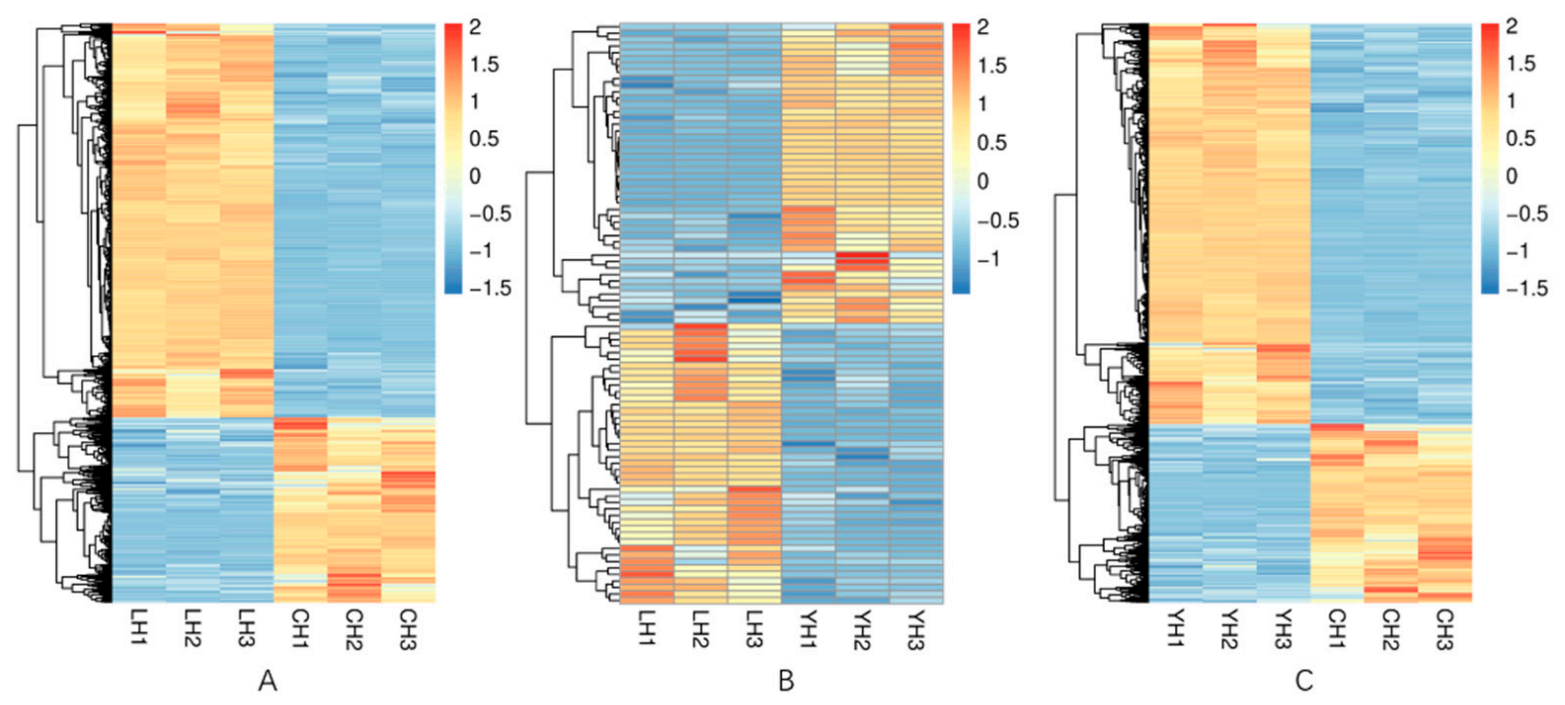
Cluster heatmaps of differentially expressed genes in the proboscis of *Apis cerana cerana* under different pollen-load conditions. Note: (**A**) LH vs. CH groups; (**B**) LH vs. YH groups; (**C**) YH vs. CH groups.

Heatmaps of DEG expression across the three comparisons (A–C) are presented in Figure 3. In all comparisons, biological replicates within each treatment group clustered together in the hierarchical clustering analysis, indicating relatively consistent expression patterns among biological replicates. For each comparison, the heatmap was generated using the DEGs identified in that specific pairwise comparison. In Heatmaps A (LH vs. CH) and C (YH vs. CH), DEGs exhibited distinct expression patterns between groups, reflecting differences in gene expression patterns between the compared groups. In contrast, Heatmap B (LH vs. YH) displayed weaker color differences and lower intergroup separation, consistent with the smaller number of DEGs identified in the LH vs. YH comparison. This suggests that workers carrying the two pollen-associated loads exhibited relatively similar overall transcriptional patterns in the proboscis.

### 3.4. Identification of Candidate Odorant Receptor (OR)-Related Genes Expressed in the Proboscis OR-like Transcripts

Transcriptome analysis of the proboscis identified six OR-like transcripts, several of which were annotated as OR-like genes. These six candidate OR-like transcripts were subjected to BLAST homology analysis and open reading frame (ORF) prediction. The results indicated that all six genes contained complete ORFs. Specifically, the longest ORF of LOC108001575 was 1173 bp and showed 99.57% similarity to *Apis cerana cerana OR9a-like* (accession number: XM_017062631.3). The longest ORF of LOC107994426 was 1017 bp and exhibited 99.85% similarity to *OR13a-like-X4* (accession number: XM_028665095.2). LOC107996752 possessed the longest ORF of 1230 bp and showed 100% similarity to *OR49b-like-X1* (accession number: XM_017054949.3). LOC107993409 had the longest ORF of 795 bp and displayed 100% similarity to *OR46a-like* (accession number: XM_017049808.3). LOC107993407 contained a longest ORF of 1119 bp and showed 100% similarity to the *Apis cerana cerana OR46a-like isoform X1* (accession number: XM_017049802.3). LOC108003486 contained the longest ORF of 1197 bp and showed 99.92% similarity to the *Apis cerana cerana* OR30a-like (accession number: XM_017065753.2).

### 3.5. Functional Enrichment Analysis

**Figure 4:**
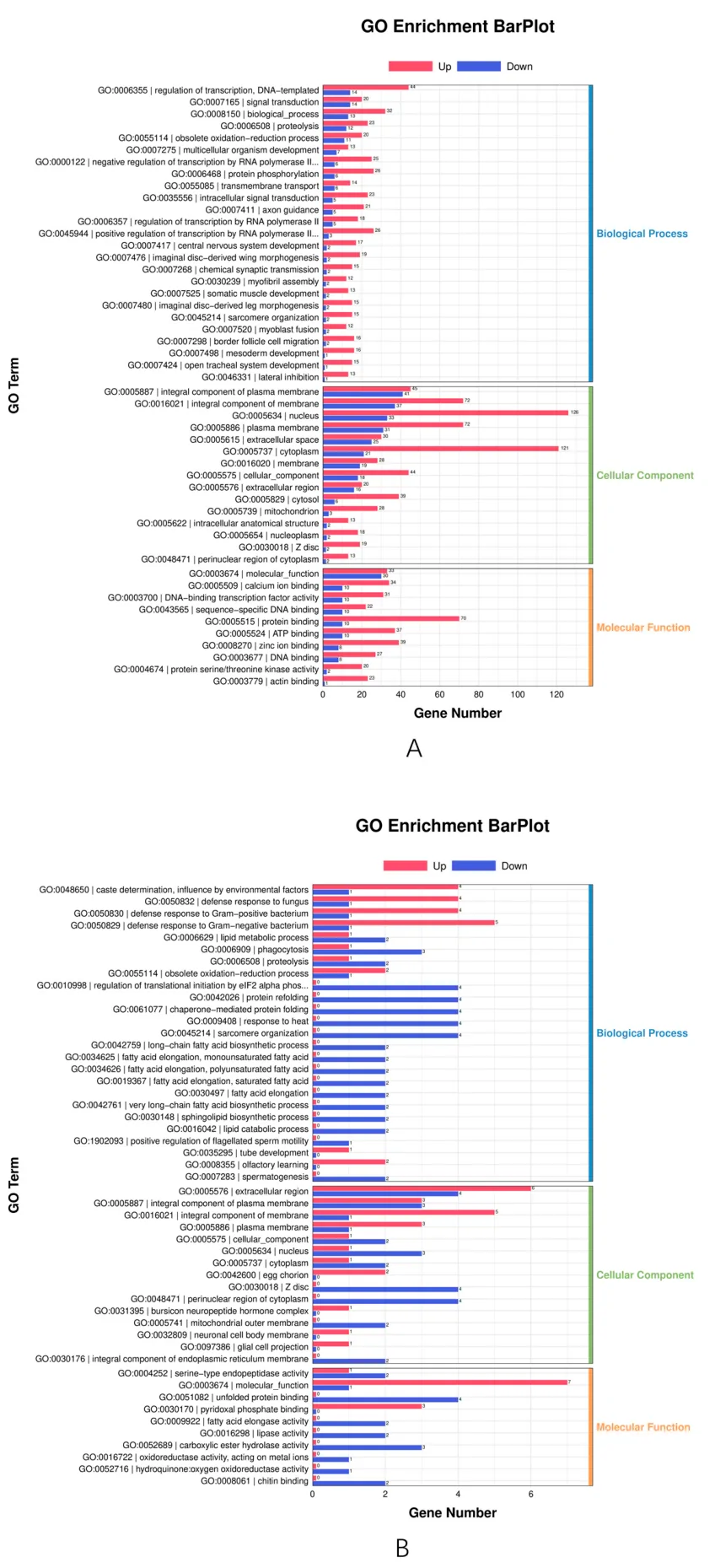

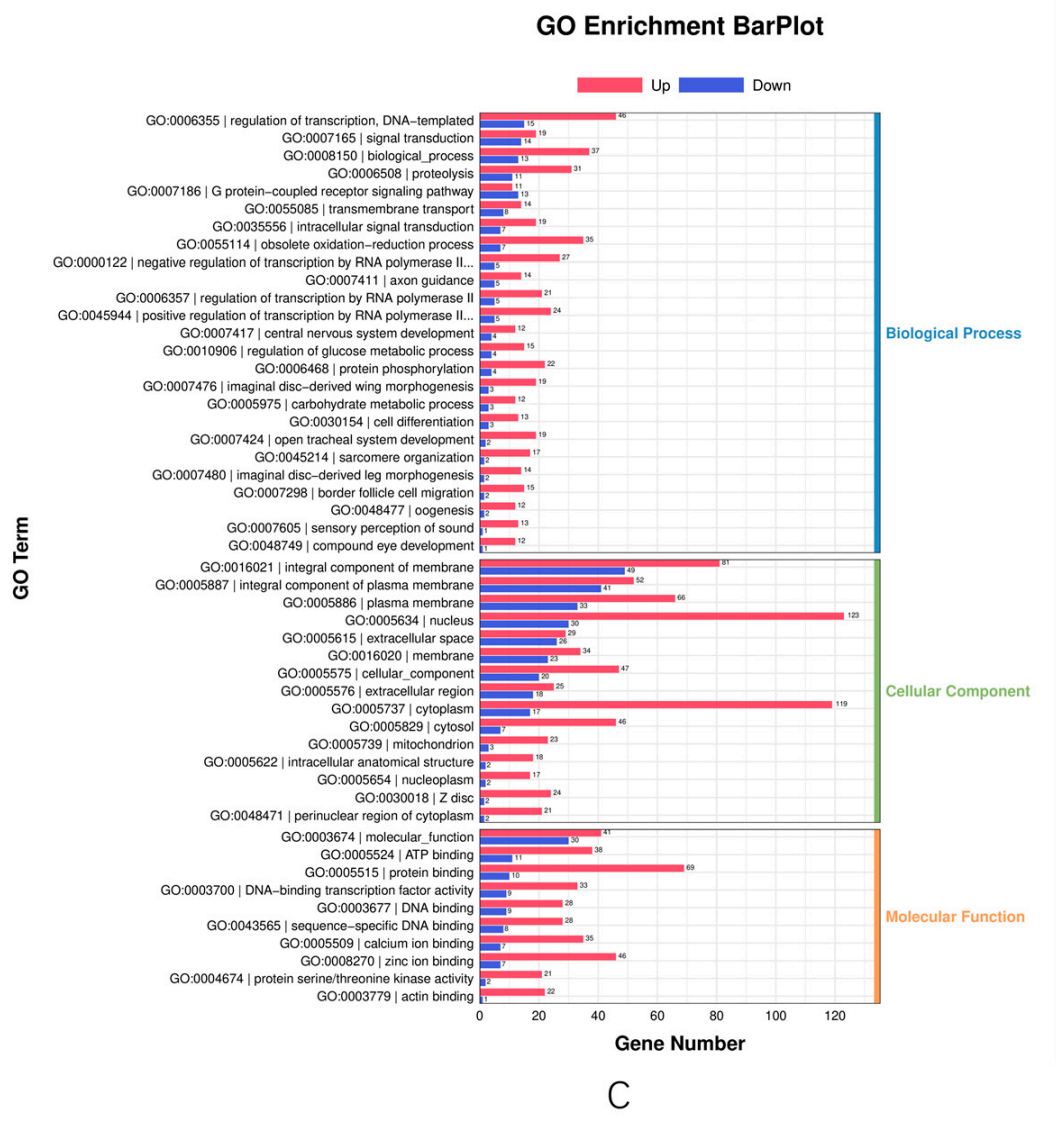
GO enrichment analysis of differentially expressed genes (DEGs) in the honey bee *Apis cerana cerana* proboscis under different pollen-load conditions. (**A**) Pear-associated pollen-load group vs. workers without visible pollen loads (LH vs. CH), (**B**) Pear-associated pollen-load group vs. Rapeseed-associated pollen-load group (LH vs. YH), and (**C**) Rapeseed-associated pollen-load group vs. workers without visible pollen loads (YH vs. CH).

The results of GO functional enrichment analysis of the three groups of differentially expressed genes (DEGs) are shown in Figure 4. The enriched terms are categorized into three major functional categories: Biological Process (BP), Cellular Component (CC), and Molecular Function (MF). Red bars represent upregulated genes, while blue bars represent downregulated genes, with bar length corresponding to the number of genes enriched in each term.

Compared with the group without visible pollen loads, upregulated DEGs in the pear-associated pollen-load group were mainly enriched in terms related to immune defense response, whereas downregulated DEGs were enriched in processes associated with olfactory perception, synapse assembly, and membrane components (Figure 4A). Between the pear-associated and rapeseed-associated pollen-load groups, upregulated DEGs were mainly enriched in defense response-related terms, whereas downregulated DEGs were enriched in terms associated with fatty acid metabolism and protein folding (Figure 4B). Compared with the group without visible pollen loads, upregulated DEGs in the rapeseed pollen group were primarily enriched in terms related to oxidation-reduction processes and lipid metabolism, whereas downregulated DEGs were enriched in terms associated with chemical signal perception and extracellular matrix components (Figure 4C). Overall, DEGs identified among the different pollen-load groups were mainly associated with functional categories related to immune response, metabolic regulation, and processes potentially related to chemical perception in the proboscis.

**Figure 5:**
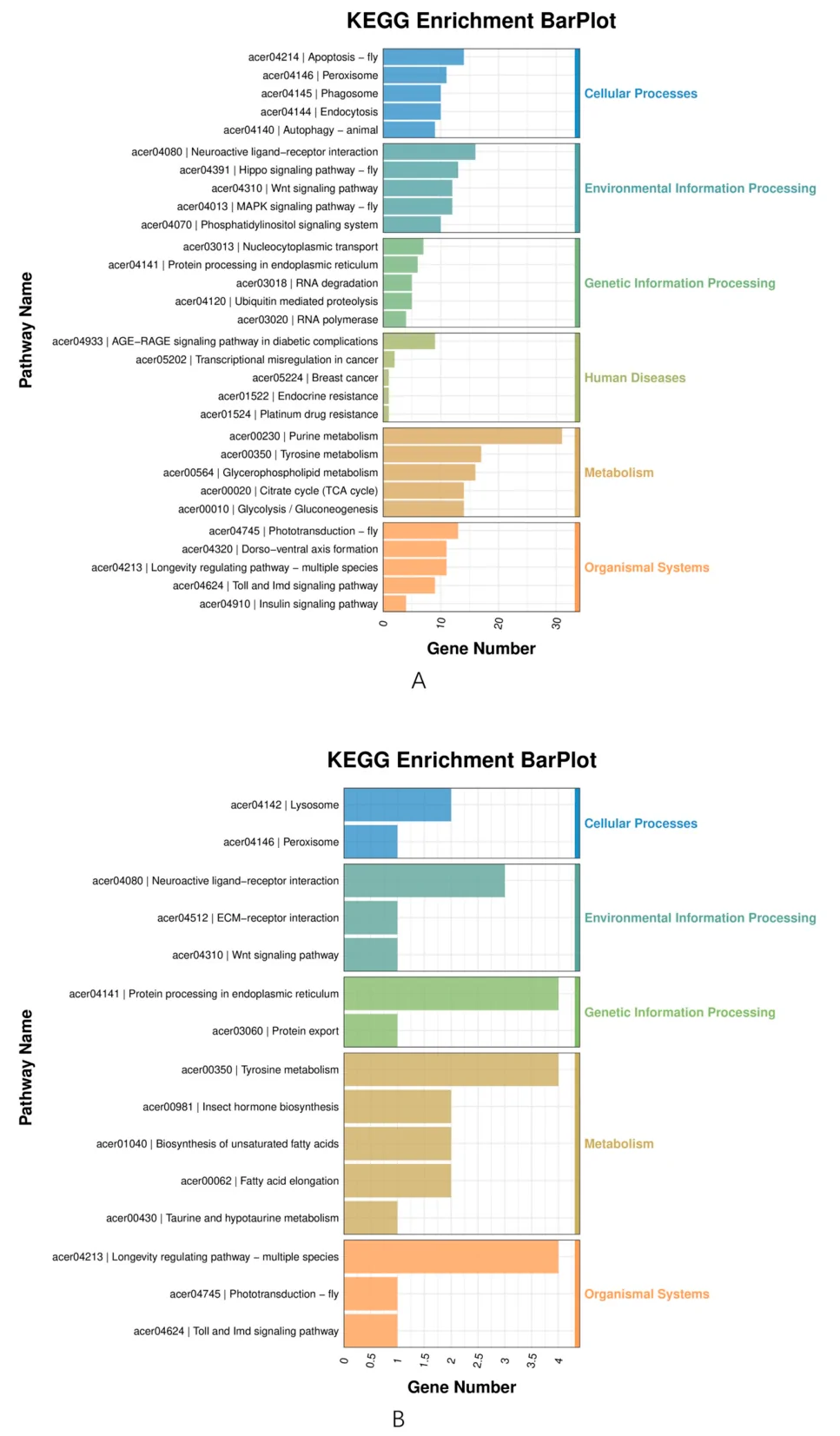

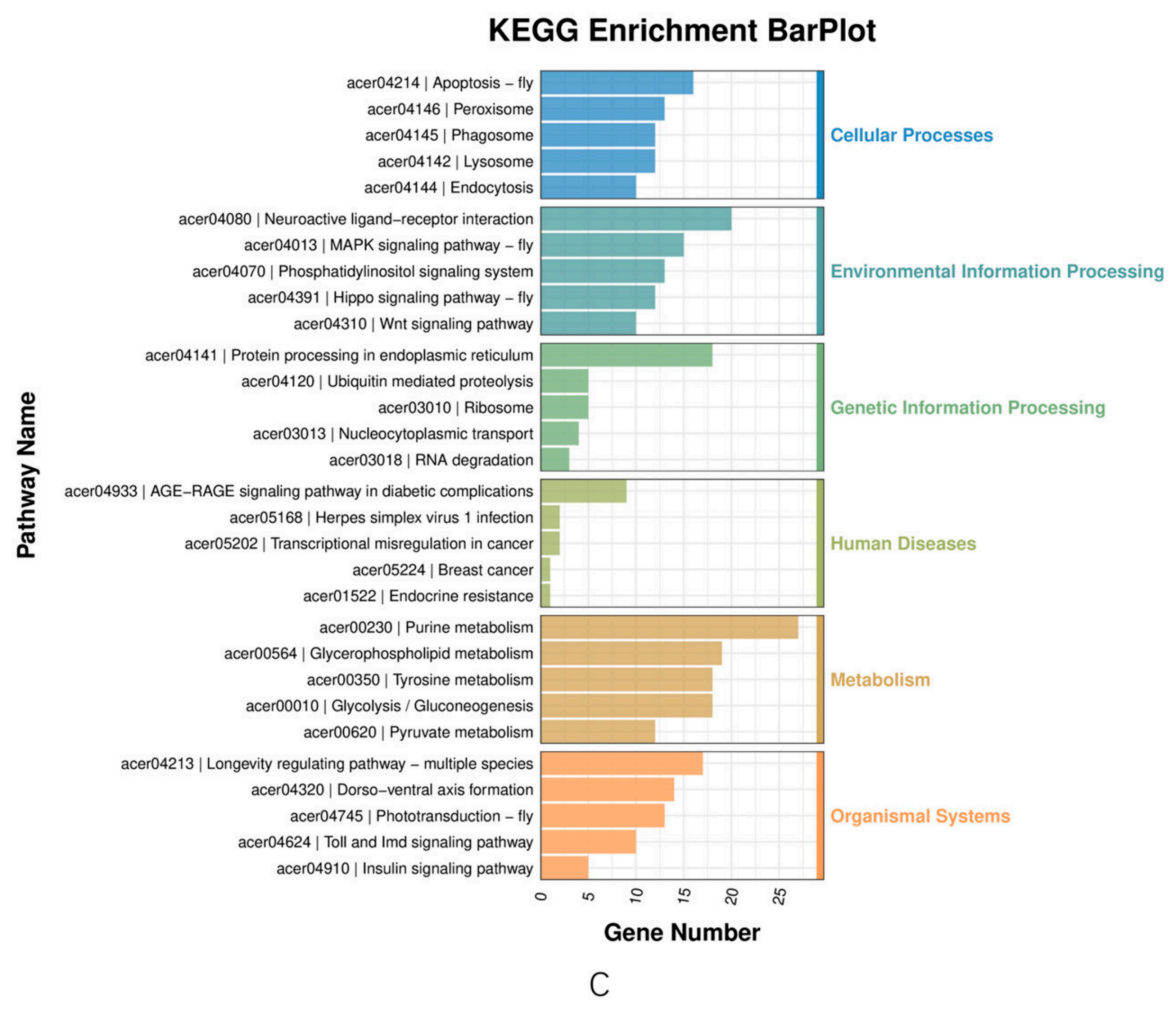
KEGG pathway enrichment analysis of differentially expressed genes (DEGs) identified from the proboscis transcriptome of *Apis cerana cerana* under different pollen-load conditions. Note: (**A**). LH vs. CH; (**B**). LH vs. YH; (**C**). YH vs. CH.

Figure 5 presents the KEGG enrichment analysis results of the differentially expressed genes. In the LH vs. CH comparison, DEGs were primarily enriched in pathways related to cellular processes and signal transduction pathways. Representative enriched pathways included lysosome, phagosome, autophagy, apoptosis, and peroxisome, as well as neuroactive ligand–receptor interaction, MAPK, Wnt, and Hippo signaling pathways. In addition, genetic information processing pathways such as protein processing in the endoplasmic reticulum, proteasome, and RNA degradation were also significantly enriched.

In contrast, the LH vs. YH comparison exhibited relatively fewer enriched pathways, mainly involving metabolism-related pathways, particularly insect hormone biosynthesis and lipid metabolism. The enrichment pattern in the YH vs. CH comparison was generally similar to that observed in LH vs. CH, with predominant enrichment in cellular homeostasis-related pathways and multiple signaling pathways.

Overall, the enrichment profiles of comparisons A (LH vs. CH) and C (YH vs. CH) were similar, whereas comparison B (LH vs. YH) showed enrichment patterns more closely associated with metabolic processes. These results suggest that the presence of pollen loads was associated with broader transcriptional differences than the differences observed between the two pollen-source groups under the conditions examined.

### 3.6. qRT-PCRValidation

**Figure 6:**
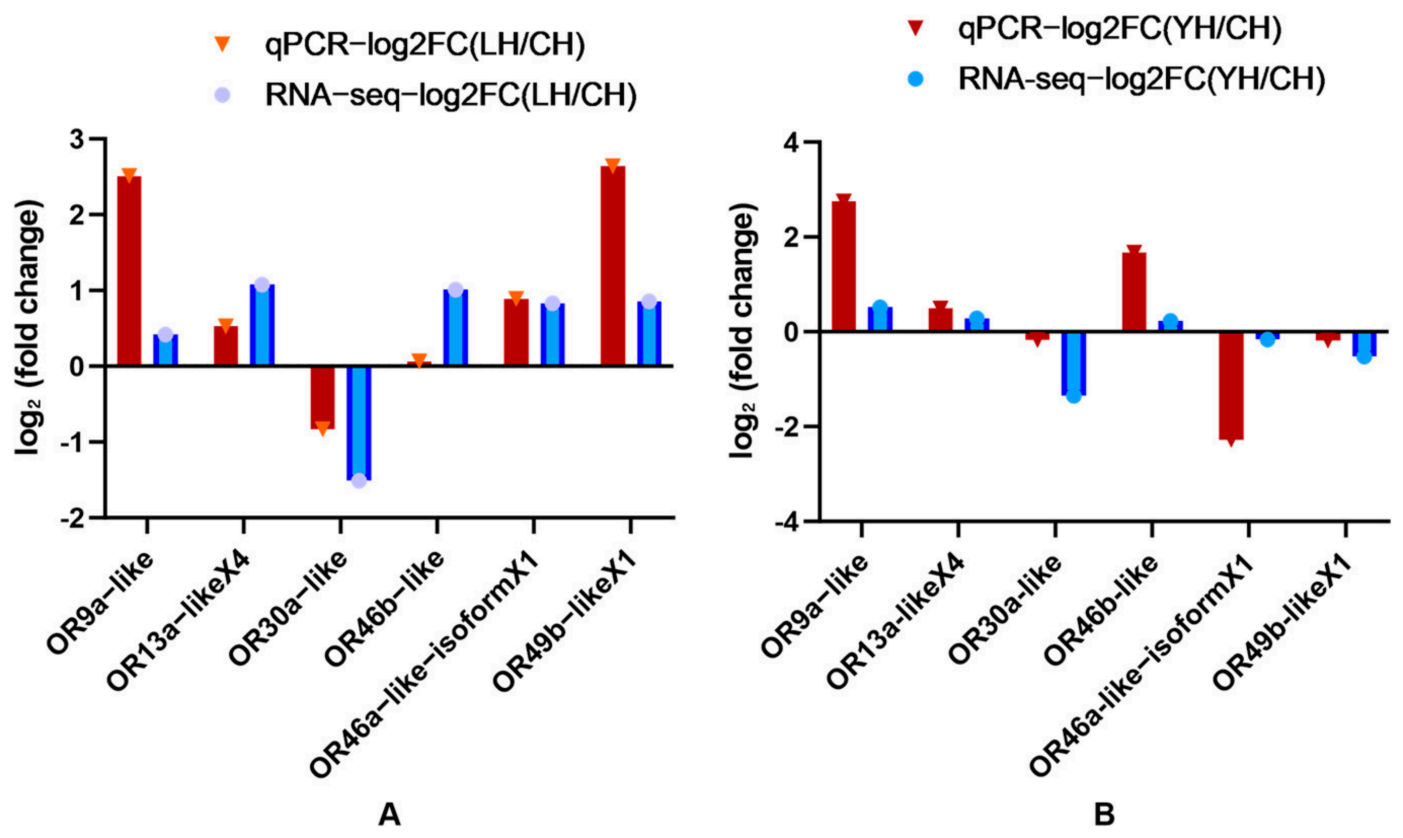
qRT-PCR validation of RNA-seq expression profiles of six candidate OR-like transcripts. Log_2_(fold change) relative to the CH group. (**A**) LH versus CH; (**B**) YH versus CH.

Six candidate odorant receptor (OR)-related genes identified from transcriptome analysis were subjected to qRT-PCR to validate RNA-seq expression patterns. The expression trends of these genes in YH, LH and CH groups were compared with RNA-seq outputs. As illustrated in Figure 6, the qRT-PCR results showed expression trends generally consistent with those obtained by RNA-seq for the six candidate OR-like transcripts. A greater magnitude of fold-change was observed in qRT-PCR assays, reflecting inherent technical discrepancies between the two detection methods.

### 3.7. Phylogenetic Analysis of AcerORs

**Figure 7:**
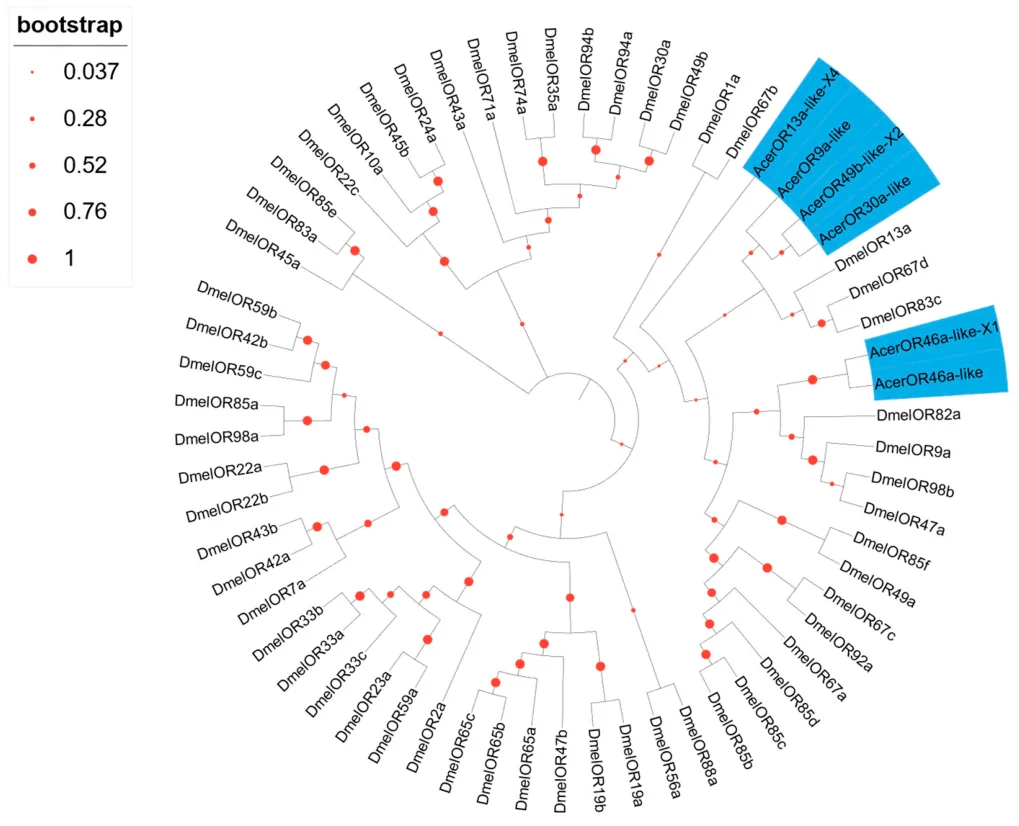
Phylogenetic tree of candidate odorant receptors AcerORs from *Apis cerana cerana* and DmelORs from *Drosophila melanogaster*.

**Figure 8:**
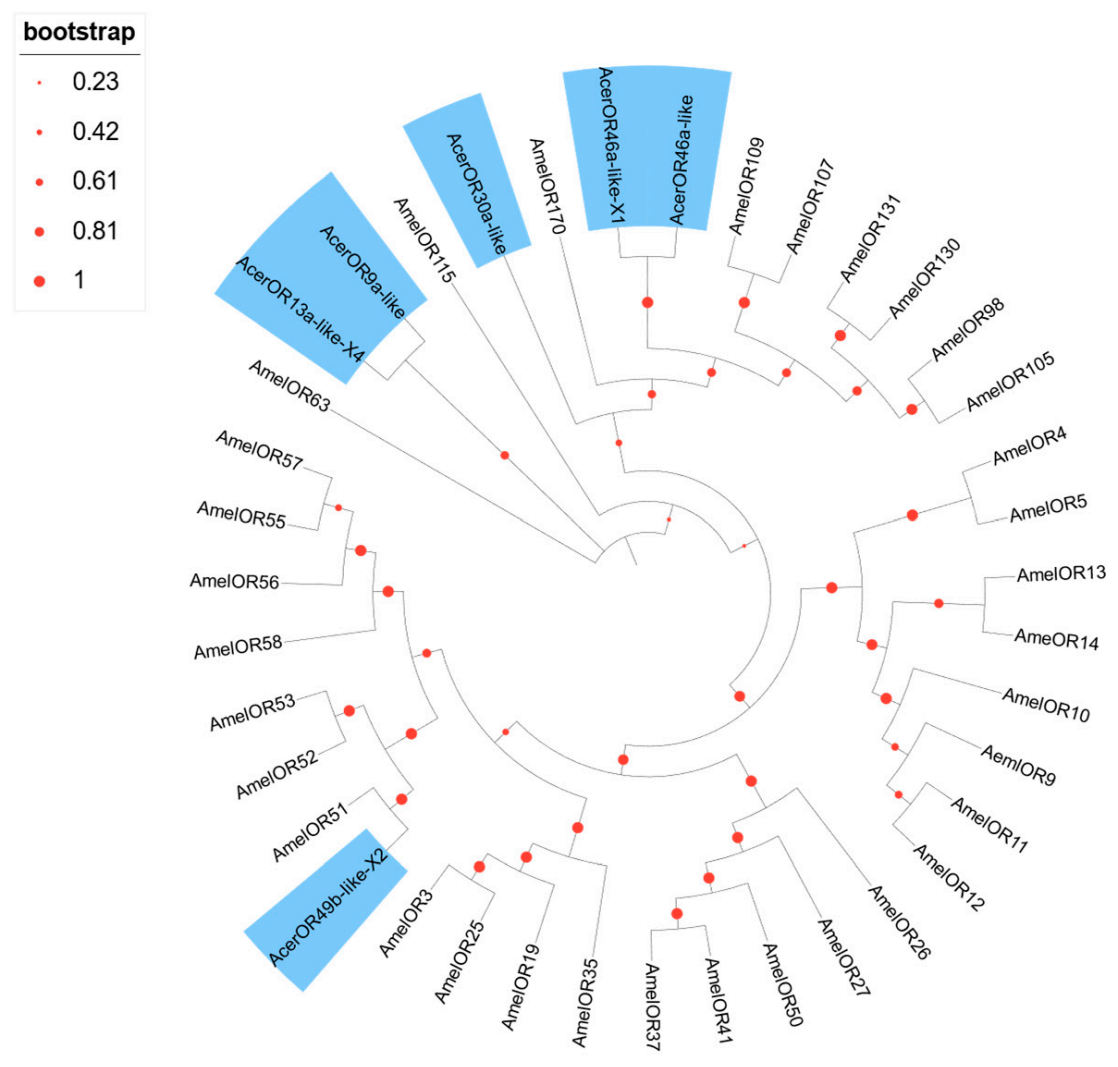
Phylogenetic tree of candidate odorant receptors AcerORs from *Apis cerana cerana* and AmelORs from *Apis mellifera*.

In the phylogenetic analysis, candidate OR sequences from *Apis cerana cerana* were used to construct circular phylogenetic trees together with annotated OR sequences from *Apis mellifera* (Amel) and *Drosophila melanogaster* (Dmel) (Figure 7 and Figure 8).

In the phylogenetic tree constructed using *Apis mellifera* reference sequences, *AcerOr46a-like-X1* clustered with *AcerOR46a-like* and was positioned close to *AmelOR109/AmelOR107*. *AcerOR30a-like* formed a clade with *AmelOR170*. *AcerOR13a-like-X4* clustered with *AcerOR9a-like.* Notably, *AcerOR13a-likeX4* was located on a relatively independent outer branch, indicating a distinct phylogenetic position among the candidate AcerOR sequences. In the phylogenetic tree constructed with *Drosophila melanogaster* reference sequences, the Acer candidate sequences similarly exhibited a clustered distribution pattern. Most candidate sequences clustered with specific DmelOR branches, whereas *AcerOR13a-like* (X4) again occupied a relatively independent branch position. Bootstrap values supporting major branches are indicated on the phylogenetic trees.

## 4. Discussion

Morphological and transcriptomic evidence collectively indicate that the distal region of the proboscis glossa in *Apis cerana cerana* represents a structurally specialized region associated with feeding activity. SEM observations showed that the distal glossa consists of the glossa and its terminal flabellum. The flabellum exhibits a fan-or brush-like morphology and contains numerous elongated, flexible hair-like sensilla [9]. Notably, sensilla appeared to be densely distributed toward the distal glossa and flabellum region. The SEM observations revealed abundant sensilla on the distal region of the proboscis, which have been suggested to participate in sensory perception in insects [13]. Consistent with this structural specialization, qRT-PCR analysis showed relatively high expression of several OR-like transcripts in the proboscis. Although the precise cellular localization of these receptors was not determined in this study, the concordance between the spatial distribution of sensilla and receptor expression supports the hypothesis that the honey bee proboscis may contain structural and molecular components associated with peripheral chemical perception. Kumar et al. identified multiple types of sensilla across various mouthpart regions in honey bees and reported that their distribution is closely associated with the regulation of liquid food intake as well as tactile and chemosensory perception [21]. Experimental evidence has demonstrated that honey bees do not rely solely on a single licking mechanism when feeding on nectar of different viscosities or sugar concentrations; instead, they can switch between licking and active suction mechanisms to enhance intake rate and optimize feeding efficiency [22]. Additionally, the kinematic properties of glossal hairs have been suggested to reduce energetic costs during feeding and influence liquid loading and recovery efficiency [23,24]. Therefore, the brush-like architecture and expanded surface area observed in the flabellum in this study may simultaneously enhance nectar contact area and carrying capacity, while increasing the sampling intensity of solutes and surface stimuli at the distal end, thereby supporting stable operation under high-frequency feeding conditions [25]. Although these structures resemble chemosensory-associated sensilla described in previous studies, their specific sensory functions cannot be determined solely based on morphology.

Importantly, the present study does not aim to demonstrate olfactory function solely based on any single line of evidence. Instead, the integration of ultrastructural observations, transcriptomic profiling, and phylogenetic analysis was intended to provide preliminary multi-level evidence regarding structural and molecular characteristics potentially associated with chemosensory processes in the proboscis. The sensilla distal glossal structures provide the morphological basis for environmental chemical contact, while the detection of several OR-related transcripts indicates the existence of molecular components potentially involved in chemical signal perception. Furthermore, the phylogenetic relationships between several candidate receptors and annotated insect OR lineages suggest evolutionary conservation among OR-related sequences. Therefore, the three analytical approaches are complementary rather than independent observations.

Transcriptome analyses showed that pollen-foraging-associated groups exhibited substantial transcriptional differences in the proboscis, whereas comparisons between different pollen sources showed comparatively limited expression divergence under the experimental conditions examined. Enrichment analyses indicated that differentially expressed genes associated with pollen-foraging conditions were related to cellular regulation, signal transduction, metabolic processes, and immune-associated pathways in the proboscis [26]. Additional enriched categories included protein processing in the endoplasmic reticulum, ubiquitin-mediated protein degradation, nucleocytoplasmic transport, multiple energy metabolism pathways, and lipid metabolism pathways. Previous studies have demonstrated the critical roles of the Toll and Imd pathways in innate immunity in honey bees and have highlighted their close association with antimicrobial effector expression and immune homeostasis maintenance [27,28,29]. The enrichment of Toll/Imd and other immune-related pathways in pollen-foraging groups suggests that changes in immune-related molecular processes may accompany differences in foraging-associated conditions [30]. Given that the proboscis directly contacts external materials during feeding, these pathways may reflect molecular responses associated with environmental exposure [31].

Although pear- and rapeseed-associated pollen-foraging bees experienced different floral environments, only 89 differentially expressed genes were identified between these two groups. In contrast, substantially larger numbers of DEGs were observed when either pollen-foraging group was compared with bees lacking visible pollen loads. This pattern suggests that differences associated with pollen-foraging status may contribute more strongly to transcriptional variation in the honey bee proboscis than differences between the two pollen sources under the present experimental conditions, whereas the influence of specific floral sources is comparatively limited under the present experimental conditions. Therefore, the floral-source-associated differences identified in this study should be interpreted as subtle transcriptional adjustments superimposed upon a broader pollen-foraging-associated molecular response. Future studies incorporating floral resources and multivariate analyses, such as principal component analysis (PCA) or variance partitioning, will help further disentangle the relative contributions of general foraging status and specific floral source to proboscis gene expression.

At the level of chemosensory-related genes, qPCR validation of six candidate odorant receptors confirmed that their expression trends were highly consistent with transcriptome results, further supporting the reliability of the sequencing data and the robustness of the differential expression analysis. In insects, OR proteins typically form heteromeric complexes with the co-receptor Orco, constituting functional ligand-gated ion channels that convert volatile stimuli into neuronal electrical signals [32,33]. At the genomic level in bees, the OR gene family has undergone significant expansion, whereas the gustatory receptor (GR) family remains relatively conserved, suggesting that volatile odor recognition may have been subjected to stronger selective pressures during bee ecological adaptation [34]. The detection of multiple OR-related transcripts in the proboscis suggests that this tissue may contain molecular components potentially associated with chemosensory perception. However, transcript detection alone is insufficient to confirm olfactory function, as receptor localization, neuronal connectivity, and functional activity remain unknown. 

Phylogenetic analysis further provided insights into the potential functional roles of candidate ORs. Most AcerORs sequences formed clusters with annotated bee ORs in the phylogenetic trees, indicating cross-species orthologous relationships. Such conserved phylogenetic correspondence generally implies high preservation of receptor structural domains and transmembrane topology, suggesting possible conservation of receptor-related structural characteristics [35]. In insects, distinct OR subfamilies often correspond to different categories of volatile compounds, including plant secondary metabolites, floral scent components, or social pheromones [36]. Therefore, phylogenetic clustering patterns suggest possible evolutionary conservation among candidate receptor OR-like transcripts, although their ligand specificity and physiological functions remain unclear.

Notably, *AcerOr13a-like* consistently occupied a relatively independent branch across different reference phylogenetic trees. Such lineage-specific branches may reflect sequence divergence among OR-like transcripts. According to molecular evolutionary theory, gene duplication provides raw material for functional innovation, and subsequent mutations may alter ligand-binding pocket structures, thereby expanding or shifting ligand spectra [37,38,39,40]. In the context of long-term adaptation of bees to diverse floral resources, the emergence of lineage-specific ORs may represent evolutionary diversification of OR-like transcripts associated with different ecological contexts [41,42,43]. Given that this gene is expressed in the proboscis and exhibits condition-responsive expression, its expression pattern and phylogenetic position make it a candidate for future functional investigation [44]. Compared with conserved branch members, receptors located in independent branches may represent divergent OR candidates worthy of further functional validation. Thus, *AcerOr13a-like* warrants priority in future functional validation studies.

Several limitations of the present study should be acknowledged. First, the identification of odorant receptor-related transcripts in the proboscis does not directly demonstrate olfactory function. Additional evidence, including spatial localization of receptor expression, electrophysiological recording, ligand-binding assays, and behavioral validation, is required to determine whether these receptors participate in volatile detection. Second, although bees were sampled based on pollen load status under different foraging conditions, several potential confounding factors could not be completely excluded, including worker age, behavioral specialization, colony-level variation, and the possibility that workers without visible pollen loads included nectar foragers, water collectors, scout bees, or other returning foragers. Consequently, the observed transcriptional differences should be interpreted as being associated with differences in pollen-foraging status rather than as evidence of floral-source-specific olfactory regulation. Future studies incorporating age-controlled and colony-controlled experimental designs together with direct behavioral observations or individual tracking will be necessary to distinguish specific foraging roles and further clarify these effects. 

Third, another important field-derived confounding factor arises from the large imbalance in floral patch size between the 54 ha pear orchard and the small 0.2 ha rapeseed fragment located 1000 m away. Under this landscape setting, rapeseed-pollen foragers (YH) constitute only a minor fraction of the local honey-bee foraging population. Bees collecting pear pollen and rapeseed pollen experience different surrounding floral volatile environments and resource landscapes, which may introduce transcriptional differences unrelated to pollen composition itself. Therefore, the 89 DEGs detected between LH and YH cannot be unequivocally attributed solely to differences in pear- versus rapeseed pollen; they may partly reflect divergent environmental backgrounds associated with the unequal size of flowering habitats. For this reason, our interpretation emphasizes the robust transcriptional contrast between pollen-carrying (LH, YH) and non-pollen-carrying (CH) bees, whereas the relatively subtle transcriptional differences between LH and YH are treated as preliminary observations. Future controlled experiments with equal-sized floral patches or caged-bee assays would help disentangle pollen-specific effects from landscape-driven environmental variation.

In summary, this study characterized the ultrastructural organization of proboscis sensilla and identified differential expression patterns of several odorant receptor-related transcripts under different pollen-foraging conditions. The integration of morphological observations, transcriptomic analysis, and phylogenetic comparison suggests that the honey bee proboscis possesses structural and molecular characteristics potentially associated with chemosensory functions in addition to its established gustatory role. These findings provide new insights into the possible involvement of peripheral mouthpart tissues in honey bee sensory ecology. However, the present study remains descriptive, and the detection of OR-related transcripts alone does not demonstrate direct olfactory function. Furthermore, potential effects of worker age, colony variation, behavioral specialization, and individual foraging history could not be completely excluded. Future studies incorporating functional validation, spatial localization of receptor expression, and more controlled experimental designs will be necessary to determine the specific contribution of the proboscis to chemical cue detection during foraging.

## 5. Conclusions

In this study, we characterized the ultrastructural features and molecular characteristics of the proboscis in *Apis cerana cerana* by integrating scanning electron microscopy, transcriptomic analysis, quantitative real-time PCR validation, and phylogenetic analysis. SEM observations revealed that the distal region of the glossa contains abundant cuticular structures, including pseudohairs and sensilla, indicating a complex surface architecture potentially associated with sensory functions during feeding.

Transcriptomic analysis demonstrated distinct gene expression patterns among bees with different pollen-foraging conditions. Differential expression and functional enrichment analyses suggested that pollen-foraging-associated states were accompanied by changes in processes related to cellular regulation, metabolism, signaling, and environmental interaction. Six candidate OR-like transcripts were identified from the proboscis transcriptome, and their expression patterns were further evaluated by qRT-PCR. Phylogenetic analysis revealed evolutionary relationships between these candidate receptors and known insect odorant receptor families, providing additional evidence for the presence of chemosensory-related molecular components in the proboscis.

Together, these findings suggest that the honey bee proboscis possesses not only feeding-related structural adaptations but also molecular features potentially associated with peripheral chemical perception. However, the current study provides primarily morphological and transcriptomic evidence, and the presence of odorant receptor transcripts alone does not demonstrate direct olfactory function. In addition, potential effects of worker age, colony variation, behavioral specialization, and individual foraging history could not be completely excluded. Future studies combining receptor localization, functional assays, behavioral experiments, and more controlled sampling designs will be necessary to clarify the specific role of the proboscis in chemical cue detection during foraging.

## Figures and Tables

**Table 1:** Primer Sequences.

Gene	Forward Primer Sequence	Reverse Primer Sequence
LOC108003486	GCTGCATATGAATGCGAGTGG	GCGGCTTCTTTGATCTTGCC
LOC108001575	TCGAGCGCAGAAACCGTTAT	AGAACGGAGAGGTAGGCCAT
LOC107994426	TTGCACATGCGTAACGTGAT	ATACACGAGCAAGCGAACCA
LOC107996752	GGTGGAATGGGAAATCAGCG	CGATTTCACTACCCTGCCCA
LOC107993409	CAGAAACCAGAGACTGGCGT	CGACGTACCAATCAGAGCGA
LOC107993407	ACTGGAGGAGCGTCTGAAAA	CGCGCAAAATTGCAAGAAGAG
*β-ACTIN*	GTTTTCCCATCTATCGTCGG	TTTTCTCCATATCATCCCAG

**Table 2:** Statistical evaluation of the proboscis transcriptome data of *Apis cerana cerana*.

Sample	Mapped Reads	Unique Mapped Reads	Raw Data Read	Valid Data Read	Valid Ratio (Reads)	Q20%	Q30%	GC Content%
CH1	26,888,315 (74.34%)	25,432,168 (70.31%)	37,003,052	36,169,928	97.75	99.98	96.72	38.50
CH2	40,454,197 (74.79%)	38,442,554 (71.07%)	55,306,598	54,089,356	97.80	99.98	97.20	38.50
CH3	387,73,069 (74.52%)	36,619,480 (70.38%)	53,190,470	52,033,296	97.82	99.98	97.04	38.50
LH1	26,906,872 (70.60%)	26,253,919 (68.89%)	394,23,744	38,109,038	96.67	99.98	97.05	40.50
LH2	27,095,512 (70.29%)	26,421,899 (68.54%)	39,847,914	38,549,130	96.74	99.98	96.72	40.50
LH3	27,115,836 (69.80%)	26,436,631 (68.05%)	40,126,482	38,850,108	96.82	99.98	96.73	40.50
YH1	31,463,706 (80.56%)	30,678,419 (78.55%)	40,477,282	39,054,488	96.48	99.98	96.85	39.50
YH2	28,822,140 (81.45%)	28,104,673 (79.42%)	36,699,442	35,386,480	96.42	99.98	96.62	39.50
YH3	30,079,153 (81.12%)	29,323,007 (79.09%)	38,437,492	37,077,560	96.46	99.98	96.48	39.50

Note: LH1–3: Three biological replicates of proboscis samples from *Apis cerana cerana* collecting pear pollen. YH1–3: Three biological replicates of proboscis samples from *Apis cerana cerana* collecting rape pollen. CH1–3: Three biological replicates of proboscis samples from workers without visible pollen loads. The same below.

## Data Availability

The RNA-seq datasets generated and analyzed during the current study have been deposited in the NCBI Gene Expression Omnibus (GEO) database under accession number GSE324143. All other data supporting the findings of this study are available from the corresponding author upon reasonable request.
